# Studying the Differences of Bacterial Metabolome and Microbiome in the Colon between Landrace and Meihua Piglets

**DOI:** 10.3389/fmicb.2017.01812

**Published:** 2017-09-21

**Authors:** Shijuan Yan, Cui Zhu, Ting Yu, Wenjie Huang, Jianfeng Huang, Qian Kong, Jingfang Shi, Zhongjian Chen, Qinjian Liu, Shaolei Wang, Zongyong Jiang, Zhuang Chen

**Affiliations:** ^1^Agro-biological Gene Research Center, Guangdong Academy of Agricultural Sciences Guangzhou, China; ^2^Brain Science Institute, South China Normal University Guangzhou, China; ^3^Ministry of Agriculture Key Laboratory of Animal Nutrition and Feed Science, Institute of Animal Science, Guangdong Academy of Agricultural Sciences Guangzhou, China

**Keywords:** microbiome, metabolome, pig breeds, colon, short chain fatty acids, bile acids

## Abstract

This study was conducted to compare the microbiome and metabolome differences in the colon lumen from two pig breeds with different genetic backgrounds. Fourteen weaned piglets at 30 days of age, including seven Landrace piglets (a lean-type pig breed with a fast growth rate) and seven Meihua piglets (a fatty-type Chinese local pig breed with a slow growth rate), were fed the same diets for 35 days. Untargeted metabolomics analyses showed that a total of 401 metabolites differed between Landrace and Meihua. Seventy of these 401 metabolites were conclusively identified. Landrace accumulated more short-chain fatty acids (SCFAs) and secondary bile acids in the colon lumen. Moreover, expression of the SCFAs transporter (solute carrier family 5 member 8, *SLC5A8*) and receptor (G protein-coupled receptor 41, *GPR41*) in the colon mucosa was higher, while the bile acids receptor (farnesoid X receptor, *FXR*) had lower expression in Landrace compared to Meihua. The relative abundances of 8 genera and 16 species of bacteria differed significantly between Landrace and Meihua, and were closely related to the colonic concentrations of bile acids or SCFAs based on Pearson's correlation analysis. Collectively, our results demonstrate for the first time that there were differences in the colonic microbiome and metabolome between Meihua and Landrace piglets, with the most profound disparity in production of SCFAs and secondary bile acids.

## Introduction

The gastrointestinal tract is a multi-function organ that harbors a dynamic microbiota population that interacts with the nutritional, physiological, and immunological functions of the host (Brestoff and Artis, 2013). Gut microbiota are influenced by many factors such as genetics, environment, diet, diseases, and lifestyle (Ananthakrishnan, 2015). Previous research suggests that genetic information of gut microbes is transmissible through generations (Goodrich et al., 2016). Furthermore, host genes related to immunity and diet could select for particular species of bacteria and archaea across different individuals, and it is possible that this effect is inherited. Support for inheritance comes from research showing that monozygotic twins possess a much more similar gut microbial community than dizygotic ones when they are raised in the same conditions (Ridaura et al., 2013).

Recent research has led to increasing recognition of the association between gut microbiota and metabolites and host physiology. Gut microbiota can influence nutrient digestion and absorption (Turnbaugh et al., 2006), lipid metabolism (Li F. et al., 2013), and hormone biosynthesis (Clarke et al., 2014) in their hosts through key functional metabolites (Clarke et al., 2014; Levy et al., 2016), which include short chain fatty acids (SCFAs), bile acids, indoles, vitamins, and polyamines (Yan et al., 2016). These metabolites can initiate various physiological and immunological responses once recognized and taken-up by host cells (Malmuthuge and Guan, 2016). For example, G protein-coupled receptors (GPR41, GPR43, and GPR109A) are activated by SCFAs to influence host physiology (Sivaprakasam et al., 2016). Microbiota-derived bile acids can modulate the metabolic activities of the host through activation of bile acid receptors such as farnesoid X receptor (FXR) and Takeda G protein-coupled receptor 5 (TGR5) (Fiorucci et al., 2009; Wahlstrom et al., 2016).

Recent studies have identified differences in the plasma and serum metabolomic profiles between two heavy pig breeds (Bovo et al., 2016), as well as differences in the colonic bacterial abundances and bacterial metabolites between fatty and lean pigs (Jiang et al., 2016). Moreover, evidence has indicated that the gut microbiomes shaped by host diet or host genotype, and can affect postnatal development of gut tissues and host metabolic health (Ha et al., 2014). Previous studies have shown the fecal microbial composition displayed diverse difference among different pig breeds (Pajarillo et al., 2014, 2015; Yang et al., 2014; Xiao et al., 2017). Microbial metabolites are major mediators linking host health, physiology, and pathology through regulation of many biological effects. However, differences in microbial communities and their metabolic activity in different pig breeds with different growth rates are largely unknown, especially in terms of how gut microbiota-derived metabolites relate to pig physiology.

Meihua is a Chinese fatty-type pig breed with a slow growth rate, while Landrace is a lean-type pig breed with a fast growth rate (Li Z. et al., 2013). In the present study, we therefore aimed to explore the differences in the colonic luminal metabolome and microbiome between Landrace and Meihua piglets by integrating taxonomic and metabolomic profiling analyses. Moreover, the relative gene expression of receptors and transporters of certain gut microbiota-derived metabolites (including SCFAs and bile acids) were determined. The results of this study may provide new insights in developing dietary intervention strategies to improve human health and animal production by manipulating host-microbiome-metabolites interactions.

## Materials and methods

### Animals and sample collection

A total of seven Landrace piglets (10.53 ± 0.52 kg initial BW) and seven Meihua piglets (3.71 ± 0.44 kg initial BW) weaned at 30 days of age were used in this study (*n* = 7). All piglets were reared under the same conditions and housed in pens with plastic slatted flooring in the research farm (Qujiang District, Shaoguan, China) of Agro-biological Gene Research Center, Guangdong Academy of Agricultural Sciences. Piglets were provided food and water *ad libitum*. Piglets were fed with the same commercial diets (Guangzhou Kingcard Biology Technology Co. Ltd., China), which contained 20.0% crude protein, 3,370 kcal of digestible energy, and 1.3% lysine. The experiment lasted 35 days. Piglets at 65 days of age were weighed again and the average daily weight gain of piglets within each breed was calculated. At the end of the experiment, all piglets were slaughtered for sample collections as previously described (Zhu et al., 2017). Fresh ileum and colon mucosa, and colon contents were collected and shock-frozen in liquid nitrogen, then stored at −80°C until analysis. All experimental procedures were carried out with the approval of the Animal Care and Use Committee in Guangdong Academy of Agricultural Sciences, China.

### Untargeted metabolomic study based on liquid chromatography tandem mass spectrometry (LC-MS/MS)

Colon contents (100 mg) from each piglet (*n* = 7) were extracted with 1,000 μL extraction solution (methanol: acetonitrile: ddH_2_O = 2: 2: 1) and 20 μL L-2-chlorophenylalanine (1 mg/mL stock in ddH_2_O, as the internal standard), and ultrasound treated for 5 min with 25 KHz intensity (SB-5200D, NingBoScientz Biotechnology Co., Ltd.). The extracted mixture was centrifuged for 15 min (16,090 g, 4°C) after incubating for 1 h at −20°C. Then, 0.5 mL supernatant was transferred to a vacuum concentrator and dried for 30 min without heating. Finally, 100 μL acetonitrile-water solution (1:1, v/v) was used to reconstitute the dry extracts, and 60 μL supernatant was transferred into a glass vial for LC-MS/MS analysis.

Two microliters of supernatant from each sample was injected into the LC-MS/MS system with HPLC (1290, Agilent Technologies) tandem TripleTOF 6600 (Q-TOF, AB Sciex) MS. The metabolome was separated with a UPLC BEH Amide column (1.7 μm, 2.1 × 100 mm, Waters). The mobile phase consisted of 25 mM NH_4_OAc and 25 mM NH_4_OH in water (pH 9.75) (A) and acetonitrile (B) and was eluted with the following gradient: 0 min, 85% B; 2 min, 75% B; 9 min, 0% B; 14 min, 0% B; 15 min, 85% B; 20 min, 85% B, which was delivered at 0.3 mL/min. A mass spectrometer (Q-TOF, AB Sciex) was used to acquire MS/MS spectra on an information-dependent basis during an LC-MS/MS experiment. In this mode, the acquisition software (Analyst TF 1.7, AB Sciex) continuously evaluated the full scan survey MS data as it collected and triggered the acquisition of MS/MS spectra depending on preselected criteria. In each cycle, six precursor ions with intensity >100 were chosen for fragmentation at a collision energy of 35 V (15 MS/MS events with product ion accumulation time of 50 ms each). Electrospray ion (ESI) source conditions were set as following: ion source gas 1 as 60, ion source gas 2 as 60, curtain gas as 30, source temperature 550°C, ion spray voltage floating 5,500 or −4,500 V in positive or negative modes, respectively.

### Metabolomic data processing and multivariate statistical analysis

The MS raw data (.d) files were converted to mzXML format using ProteoWizard, and processed using the R (version 3.3.2) package XCMS. The preprocessing results generated a data matrix that consisted of the retention time (RT), mass to-charge ratio (m/z) values, and normalized peak intensity. 3,864 and 3,840 peaks were detected under LC-MS (ESI+) and LC-MS (ESI-) after pre-processing the detected signals, respectively. The R package CAMERA and an in-house database were used for peak annotation after XCMS data processing (Wang et al., 2016). Further, multivariate statistical analyses were conducted using the SIMCA software package (V14, Umetrics AB, Umea, Sweden) on the resulting three-dimensional data matrix.

After the data matrix was mean-centered and scaled to the pareto variance, principal components analysis (PCA) and orthogonal partial least squares-discriminant analysis (OPLS-DA) were carried out to generate an overview of the variables of colon contents and to visualize the differences between pig breeds, respectively (Wheelock and Wheelock, 2013). For model diagnosis, an appropriate OPLS-DA model must meet at the condition of *P*-value (CV-ANOVA) < 0.05. In addition, the parameters R^2^Y and Q^2^ are used to evaluate the predictive ability and fitting level of the model resulting from internal validation. After model diagnosis, metabolites for separating the models were selected with the following requirements: variable importance in the projection (VIP)>1 and |p(corr)| ≥ 0.5 with 95% jack-knifed confidence intervals. The Student's *t*-test was applied to further analyze intergroup significance of the selected metabolites.

### SCFA quantification by gas chromatography-mass spectrometry (GC-MS)

The extraction and quantification of SCFAs in the colon contents were performed as previously described (Sun et al., 2017).

### Bile acid quantification by LC-MS/MS

Colon content from piglets of two breeds (*n* = 7) was extracted as previously reported (Cai et al., 2012), with some modifications. Briefly, 100 mg of colon content for each sample was extracted with 1 mL of 10 mM ammonium acetate in 70% methanol followed by vortexing for 30 s twice before and after shaking for 20 min, then centrifuging at 13,000 g at 4°C for 10 min. Finally, 200 μL of extract solution was used for quantification by LC-MS/MS. The bile acid extract solution (10 μL) was injected into a C18 column (AQUITY UPLC BEH 130, 1.7 μm, 2.1 by 100 mm, Waters) at a flow rate of 0.1 mL/min, and the column temperature was maintained at 40°C. The solution was separated by reversed phase ultra-fast LC (Shimadzu, Kyoto) with a multi-step linear gradient elution using solution A (10 mM ammonium acetate–ammonium hydroxide buffer at pH 8.0) and solution B (10 mM ammonium acetate in acetonitrile–methanol solution, 3:1) over 30 min. The gradient profile was set as follows: 30–65 % B over 6 min; 65–72% B over 8 min; 72–90% B over 1 min; 90–90% B over 5 min; 90–30% B over 0.1 min. Then the column was equilibrated with 30% mobile phase B for 10 min. The eluate was then introduced into the ESI source of a tandem triple quadrupole MS analyzer (API4000, AB Sciex, Foster City, CA), and cholic acid (CA), deoxycholic acid (DCA), chenodexycholic acid (CDCA), and lithocholic acid (LCA) authentic compounds were quantified in multiple reaction monitoring (MRM) mode using optimized MS/MS conditions (Table S1). MS conditions were as follows: source, Turbo IonSpray; ion polarity, negative; IonSpray voltage, 4,500 V; source temperature, 550°C; gas, nitrogen; curtain gas, 25 psi; nebulizing gas (GS1), 55 psi; collision gas (GS2), 55 psi; scan type, MRM; Q1 resolution: unit; Q3 resolution: unit. Analyst 1.5.2 software (AB Sciex, Foster City, CA) was used to control the instrument and to acquire and process all MS data.

### Gene expression study by quantitative real-time PCR (qRT-PCR)

Total RNA was extracted from frozen colon and ileum mucosal tissues (*n* = 3) using TranzolUp reagent (TransGen Biotech, China) according to the manufacturer's instructions. The concentration and quality of extracted total RNA were determined by a NanoDrop-ND2000 spectrophotometer (Thermo Fisher Scientific Inc., Germany). The integrity of total RNA was further checked by gel electrophoresis on a 1% agarose gel for visualization of complete 28 and 18S bands. Genomic DNA was eliminated by treatment with DNase I (TransGen Biotech, China). Complementary DNA (cDNA) was then synthesized from 1 μg of total RNA using an M-MLV First Strand Kit (Invitrogen, USA) following the instructions of the manufacturer. The qRT-PCR for gene expression was performed in duplicate using a ChamQTMSYBR®qPCR Master Mix (Vazyme Biotech Co., China) on a CFX connect system (Bio-Rad, USA). Specificity of the amplification was confirmed by the melting curve. Primers were designed using Primer Premier 5.0 software (Applied Biosystems, USA) and were synthesized by Generay Biotech Co. (Shanghai, China). Primer sequences, annealing temperatures (Tm), and product lengths of target genes are listed in Table S2. The fold change of the target genes was normalized to housekeeping gene (β-actin) and was calculated using the 2^−ΔΔCT^ method.

### ATP quantification by HPLC

Colon content (25 mg) from piglets of two breeds (*n* = 7) was extracted with 1 mL of 0.3 M HClO_4_, and sonicated once for 5 min after vortex mixing, followed by centrifugation for 5 min (16,090 g, 4°C). The supernatant (160 μL) was then transferred to a new tube containing 2 M KOH solution and equilibrated at 4°C for 3 h. After centrifugation (5 min, 16,090 g, 4°C), the supernatant was passed through a membrane filter (0.22 μm), and 150 μL was transferred into a 2 mL glass vial for HPLC analysis. HPLC analysis was performed using methanol: 50 mM KH_2_PO_4_ buffer solution (9:91, v:v) (pH 6.5) as the mobile phase with a flow rate of 0.5 mL/min, and detected using a UV detector at a wavelength of 259 nm. Finally, data processing was performed using Chromeleon version 6.8 (Thermo Fisher).

### Bacterial DNA extraction, PCR amplification, high-throughput sequencing, and bioinformatics analysis

Microbial genomic DNA from piglets of two breeds (*n* = 7) was extracted from 200 mg of each colonic sample using QIAamp DNA stool minikit (Qiagen, Germany) according to the manufacturer's instructions. 2.5 μL diluted DNA sample (5 ng/μL) was used for 25 μL PCR reaction mixtures. 10 μL of primers (forward primer, 341F 5″-CCTACGGGAGGCAGCAG-3″; reverse primer 806R 5″-GGACTACHVGGGTWTCTAAT-3″ with attaching12 bp barcode sequences) at 1 μM concentration were used to amplify a portion of the V3–V4 region of bacterial 16S rRNA genes with 12.5 μL TaKaRa ExTaq polymerase mixtures. PCR amplicon products were purified using AMPure XP beads (Biomek, USA) and were checked for quality on an Agilent 2100 bioanalyzer (Agilent, USA). Amplicons were paired-end sequenced on the Illumina MiSeq platform using 2 × 250 bp MiSeq reagent kit v3 (Illumina, USA). Raw reads were submitted to the Sequence Read Archive of the NCBI (accession number SRP 095863).

In order to obtain high quality sequences, head or tail bases with qualities lower than Q30 were trimmed, and sequence lengths shorter than 100 bp were removed. Fastq-join (v1.3.1) (Aronesty, 2013) was used to combine paired-end reads. Assembled sequences were analyzed with QIIME software (v1.8.0) (Caporaso et al., 2010) to obtain operational taxonomic units (OTU) using the closed-reference OTU picking method with default OTU clustering tool UCLUST (uclust v1.2.22) such that each clustered OTU was at the 97% similarity level. Representative sequences of OTUs were selected based on the maximum length and were aligned to Greengenes 16S rRNA gene database (v13.8) with the RDPII classifier (v2.2) (Wang et al., 2007) to obtain taxonomic assignments. For species level identification, all OTUs were aligned to bacterial genome sequences in GenBank using the BLAST (Basic Local Alignment Search Tool) algorithm (blast v2.2.25) and parsed with the following criteria:(1) best hit; (2) cutoffs of 90% identity and 400 bp alignment length; (3) in accordance with the axonomic assignments from QIIME. Alpha-diversity indices (Chao1, Shannon, PD whole tree, and observed species) were calculated based on a subset of randomly selected sequences from each sample. Beta-diversity of weighted UniFrac-based PCoA (principal coordinate analysis) was calculated to show the group differences. Beta-diversity statistical analyses were tested using PERMANOVA based on Bray-Curtis dissimilarities and 999 permutations in R (v3.2.0). Microbial function was predicted using PICRUSt (Phylogenetic Investigation of Communities by Reconstruction of Unobserved States) (v1.1.0) (Langille et al., 2013). Differences among groups were compared with the software STAMP (Parks et al., 2014). Two-side *t*-test and Benjamini-Hochberg FDR correction from STAMP were used in two-group analysis. Differences were considered significant at *P* < 0.05. The correlation coefficients between metabolic compounds and bacterial compositions at both genus and species level were calculated using Pearson's correlation test in R software (v3.2.2).

## Results

### Metabolic differences of gut microbiota between Landrace and Meihua piglets

**Figure 1:**
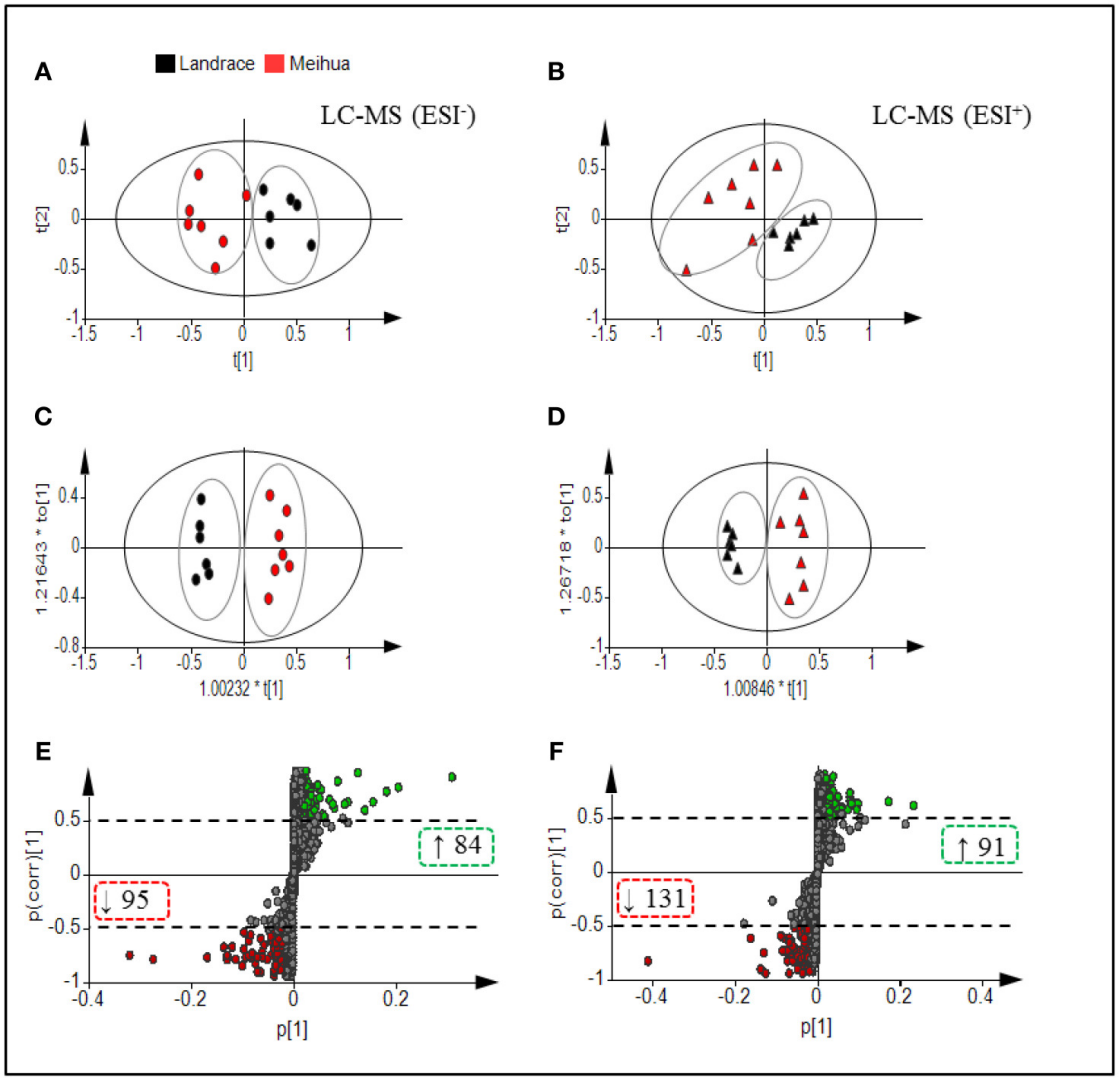
Multivariate statistical analysis of untargeted metabolomic data obtained using the LC-MS/MS approach. PCA Score plot of colonic metabolomic data for Landrace (black) and Meihua (red) piglets obtained by **(A)** LC-MS (ESI−) and **(B)** LC-MS (ESI+). **(C)** OPLS-DA Score plot of colonic metabolomic data obtained by LC-MS (ESI−); R^2^Y = 0.976, Q^2^ = 0.816; and *P*(CV-ANOVA) = 0.0049. **(D)** OPLS-DA Score plot of colonic metabolomic data obtained by LC-MS (ESI+) data; R^2^Y = 0.963, Q^2^ = 0.73 and *P* (CV-ANOVA) = 0.021. **(E)** S-plot of LC-MS (ESI-) data with 3,480 metabolite signals detected. **(F)** S-plot of LC-MS (ESI+) data with 3,864 metabolite signals detected. Red circles in S-plots are model-separated metabolites following the conditions of VIP >1 and | *P* (corr)| ≥ 0.5 with 95% jack-knifed confidence intervals. Red or green rectangles in S-plots identify the numbers and tendency of metabolites to separate in the model when Meihua piglets are compared with Landrace piglets.

In the PCA score-plots (Figures 1A,B), the two pig breeds were distributed in separate groups based on the first two principal components, which indicate significant differences in the metabolites between them. The score-plot of OPLS-DA, a method of supervised pattern recognition, further revealed that the colonic metabolome of Meihua piglets could be clearly distinguished from that of Landrace piglets (Figures 1C,D). Parameters for evaluating the predictive ability and fitting level of the model resulting from internal validation suggested that the three models possessed a satisfactory fit with good predictive power. These parameters were: values of R^2^Y and Q^2^ > 0.7, and *P*-values of CV-ANOVA < 0.05, which meant the groups within the models were reliable and significantly different (Figures 1C,D). Further, based on a VIP > 1 in 95% jack-knifed confidence intervals and |*P*(corr)| ≥ 0.5, a total of 222 and 179 biomarker metabolites, which were detected in the LC-MS/MS (ESI+) and LC-MS/MS (ESI-) respectively, were selected according to the OPLS-DA S-plot (Figures 1E,F).

In total, relative levels of 401 biomarker metabolites differed significantly between Landrace and Meihua piglets. Detailed information about the 401 biomarker metabolites is in Data S1. In-depth analyses using an in-house MS/MS database established by authentic standards allowed for conclusive identification of 70 biomarker metabolites (Table 1). Those 70 compounds included bile acids, free amino acids, dipeptides, lipids, nucleotides, organic acids, nitrogen-containing compounds, and so on. In Landrace piglets, the relative levels of five bile acids (deoxycholic acid, lithocholic acid, glycocholate, ursodeoxycholic acid, and taurolithocholic acid), four sphingolipids [Lyso PE (20:3n6/0:0), PE (20:3/0:0), phytosphingosine, and sphinganine], two fatty acids (stearic and 16-hydroxypalmitic acid), four benzene derivatives (hydroquinone, 3-methylphenyacetic acid, 1,4-dihydroxybenzene and 3-hydroxybenzoate), and 11 other compounds in the colon lumen were significantly higher than those in Meihua piglets (*P* < 0.05). However, the relative levels of six free amino acids (glutamine, glutamate, D-proline, L-proline, citrulline, and tyrosine), eight dipeptides (Phe-Thr, Lys-Leu, Pro-Val, IIe-Pro, His-IIe, Lys-Pro, and Phe-Val), five nucleotides (thymine, uracil, adenine, deoxyguanosine, and 2-hydroxyadenine), and 16 other compounds were significantly lower in Landrace piglets compared to Meihua piglets (*P* < 0.05).

**Table 1:** Identified metabolites for discriminating between Landrace and Meihua piglets based on the untargeted metabolomics study.

**Metabolic pathway**	**RT^a^ (min)**	**Detection mode**	**VIP**	**p (corr)**	**Mean-Landrace**	**Mean-Meihua**	***t*-test^b^**
**BILE ACIDS METABOLISM**							
Deoxycholic acid	6.522	LC-MS ESI-	9.6825	0.76131	1.56E−02	5.55E−03	^**^
Lithocholic acid	5.988	LC-MS ESI+	2.05897	0.61714	4.42E−04	6.17E−05	^*^
Taurolithocholic acid	7.772	LC-MS ESI+	1.05204	0.81984	0.000142	0.0000724	^***^
Glycocholate	4.721	LC-MS ESI-	1.39793	0.803073	0.00029897	0.00010597	^**^
Ursodeoxycholic acid	4.71187	LC-MS ESI+	1.75393	0.812378	0.000297555	0.0000774	^**^
**DIPEPTIDES**							
Phe-Thr	3.852	LC-MS ESI+	1.14398	0.69232	0.0000206	0.000125	^*^
Lys-Leu	4.337	LC-MS ESI+	1.35231	0.66845	0.000128	0.000287	^***^
Pro-Val	3.586	LC-MS ESI+	1.27253	0.61089	0.0000627	0.000205	^*^
Ile-Pro	3.82	LC-MS ESI+	3.29598	0.68484	0.000668	0.00149	^**^
His-Pro	3.919	LC-MS ESI+	1.49293	0.60608	0.00013	0.000337	^*^
Phe-Pro	4.386	LC-MS ESI+	1.87255	0.54983	0.000369	0.00072	
His-Ile	8.80625	LC-MS ESI+	1.35118	0.73756	8.55865E−05	0.00020965	^**^
Lys-Pro	14.627	LC-MS ESI+	2.1594	0.799666	0.000314194	0.00062943	^**^
Phe-Val	8.19602	LC-MS ESI+	1.00549	0.696012	0.00001605	0.0000976	^*^
**ORGANIC ACIDS METABOLISM**							
2-Butenoic acid	1.684	LC-MS ESI+	3.80601	0.57964	0.00147	0.00286	
Dodecanoic acid	3.32	LC-MS ESI-	1.07499	0.66646	0.00025	0.000392	^*^
Alpha-Linolenic acid	1.14592	LC-MS ESI-	7.29765	0.934984	0.00935933	0.0139623	^***^
16-Hydroxypalmitic acid	4.521	LC-MS ESI-	6.0069	0.85129	0.00384	0.000565	^***^
Stearic acid	1.38912	LC-MS ESI+	1.65239	0.744532	0.000699651	0.00049507	^**^
4-Guanidinobutyric acid	10.7132	LC-MS ESI+	2.07103	0.593309	0.000571072	0.00101013	
3-Hydroxybutyric acid	4.118	LC-MS ESI+	1.28956	0.59916	0.000142	0.000296	^*^
Levulinic acid	4.169	LC-MS ESI+	2.5701	0.6198	0.000219	0.000835	^*^
Succinate	1.95	LC-MS ESI-	1.10273	0.55747	0.000175	0.00067	^*^
**SPHINGOLIPID METABOLISM**							
LysoPE (20:3n6/0:0)	8.405	LC-MS ESI+	1.00886	0.71241	0.000117	0.000046	^**^
PE (20:3/0:0)	8.039	LC-MS ESI+	3.80258	0.74022	0.0017	0.000719	^**^
Phytosphingosine	5.005	LC-MS ESI+	1.59896	0.79725	0.000224	0.000061	^***^
Sphinganine	5.038	LC-MS ESI+	2.26242	0.74951	0.000463	0.000108	^**^
**ARGININE AND PROLINE METABOLISM**							
Glutamate	2.434	LC-MS ESI-	1.01355	0.51585	0.000458	0.000603	^*^
Glutamine	2.451	LC-MS ESI+	1.04735	0.61003	0.000143	0.00023	^*^
5-Aminopentanoic acid	1.968	LC-MS ESI+	5.46205	0.62789	0.00168	0.00427	^*^
D-Proline	9.50385	LC-MS ESI+	2.95699	0.593271	0.000792095	0.00157409	^*^
L-Proline	9.5367	LC-MS ESI+	1.89764	0.515471	0.000137675	0.00050401	
N-Acetylputrescine	2.185	LC-MS ESI+	2.55803	0.60163	0.000572	0.00124	
Citrulline	2.335	LC-MS ESI+	1.20243	0.66665	0.000295	0.000184	^**^
N-Acetylputrescine	10.1657	LC-MS ESI+	2.55803	0.601628	0.000572109	0.00123815	
N-Acety-L-glutamate	3.134	LC-MS ESI-	1.65456	0.70721	0.00009	0.000502	
**TYROSINE METABOLISM**							
Tyrosine	3.035	LC-MS ESI+	1.40856	0.75924	0.000173	0.000313	^**^
Tyramine	2.001	LC-MS ESI+	4.91784	0.72541	0.00143	0.00322	^**^
4-Methoxyphenylacetic acid	5.519	LC-MS ESI-	1.14392	0.53676	0.000119	0.000328	
Hydroquinone	1.03603	LC-MS ESI-	1.99776	0.610798	0.000521502	0.0000483	^*^
3-Methylphenylacetic acid	2.484	LC-MS ESI-	8.00808	0.68299	0.00928	0.0018	^*^
1,4-Dihydroxybenzene	1.817	LC-MS ESI-	1.08224	0.65869	0.000224	0.0000857	^*^
3-Hydroxybenzoate	3.367	LC-MS ESI+	1.67021	0.67459	0.00121	0.00097	^*^
Dopamine	2.268	LC-MS ESI+	1.32803	0.74421	0.000145	0.000272	^**^
**PYRIMIDINE METABOLISM**							
Cytosine	1.868	LC-MS ESI+	2.17035	0.81631	0.000729	0.000396	^**^
Cytidine	4.068	LC-MS ESI+	1.44061	0.79162	0.000326	0.000176	^**^
Thymine	2.084	LC-MS ESI-	1.57467	0.71573	0.000215	0.000502	^*^
Uracil	1.85	LC-MS ESI-	4.91099	0.86249	0.00181	0.00408	^***^
Adenine	2.234	LC-MS ESI-	4.31482	0.66043	0.000225	0.00261	^*^
Deoxyguanosine	4.435	LC-MS ESI-	1.10168	0.53988	0.000123	0.00029	^*^
2-Hydroxyadenine	2.534	LC-MS ESI+	3.75296	0.53183	0.00105	0.00224	^*^
**OTHERS**							
O-Acetylcholine	11.3929	LC-MS ESI+	5.50725	0.597001	0.00689022	0.00420787	^*^
D-Lyxose	4.43453	LC-MS ESI-	1.55	0.632079	0.000296192	0.00061133	^*^
Glutaraldehyde	1.384	LC-MS ESI+	1.26127	0.60349	0.000153	0.000299	^*^
Glycyrrhetinic acid	1.28742	LC-MS ESI-	1.96603	0.639127	0.000175096	0.00067003	^*^
L-Carnitine	2.702	LC-MS ESI+	4.59919	0.64673	0.00116	0.00309	^*^
Ketoisocaproic acid	2.151	LC-MS ESI-	4.03199	0.69535	0.000726	0.0026	^*^
2-Oxoadipic acid	2.667	LC-MS ESI-	1.01241	0.8472	0.000114	0.000214	^***^
Beta-Glycyrrhetinic acid	7.822	LC-MS ESI-	1.96603	0.63913	0.000175	0.00067	^*^
2-Indolecarboxylic acid	7.71052	LC-MS ESI+	2.03021	0.545726	0.000760285	0.00039665	^*^
Norvaline	1.968	LC-MS ESI+	2.54301	0.57594	0.000856	0.000268	^*^
Oleanolic acid	7.589	LC-MS ESI-	1.64358	0.68042	0.0156	0.00555	^**^
p-Chlorophenylalanine	3.334	LC-MS ESI+	3.43195	0.69057	0.00429	0.00329	^*^
Betaine aldehyde	2.702	LC-MS ESI+	2.65383	0.5971	0.00105	0.000471	^*^
Picolinic acid	2.067	LC-MS ESI+	1.56513	0.74737	0.000282	0.000105	^**^
Pyrrolidine	1.201	LC-MS ESI+	3.27502	0.71124	0.0011	0.000269	^**^
Erucamide	5.64	LC-MS ESI+	1.50832	0.65965	0.000702	0.000507	^*^
Nicotinamide	2.051	LC-MS ESI+	3.28287	0.52826	0.00127	0.000147	
3-Mercapto-2-butanone	8.95645	LC-MS ESI+	1.43044	0.761824	0.000148586	0.00029097	^**^
1H-Purin-6-amine, N,N-dimethyl-Aminobutyrate	1.92688	LC-MS ESI+	1.61983	0.588964	0.0000449	0.00026979	^*^

*RT^a^, Retention time. t-test^b^, Student t-test of the relative levels of compounds detected in the colon lumen of Landrace piglets and Meihua piglets (n = 7)*.

* *p < 0.05*,

** p < 0.01 and

*** *p < 0.001, respectively*.

*Asterisks in red were those metabolites with higher relative levels in Landrace piglets compared with Meihua piglets, otherwise the levels were lower in Landrace piglets (n = 7)*.

The metabolome view map generated by the software Metaboanalyst 3.0 revealed that nine relevant pathways were significantly enriched for those 70 metabolites based on *P*-value (<0.05) or impact value (>0.1) (Figure S1). The impact values for those seven pathways, including arginine and proline metabolism, pyrimidine metabolism, alanine-aspartate-glutamate metabolism, tyrosine metabolism, D-Glutamine and D-glutamate metabolism, sphingolipid metabolism, and pantothenate-CoA biosynthesis, were 0.199, 0.164, 0.384, 0.152, 0.139, 0.140, and 0.180, respectively (Table S3).

### High levels of SCFAs and secondary bile acids accumulated in the colon lumen of Landrace piglets

**Figure 2:**
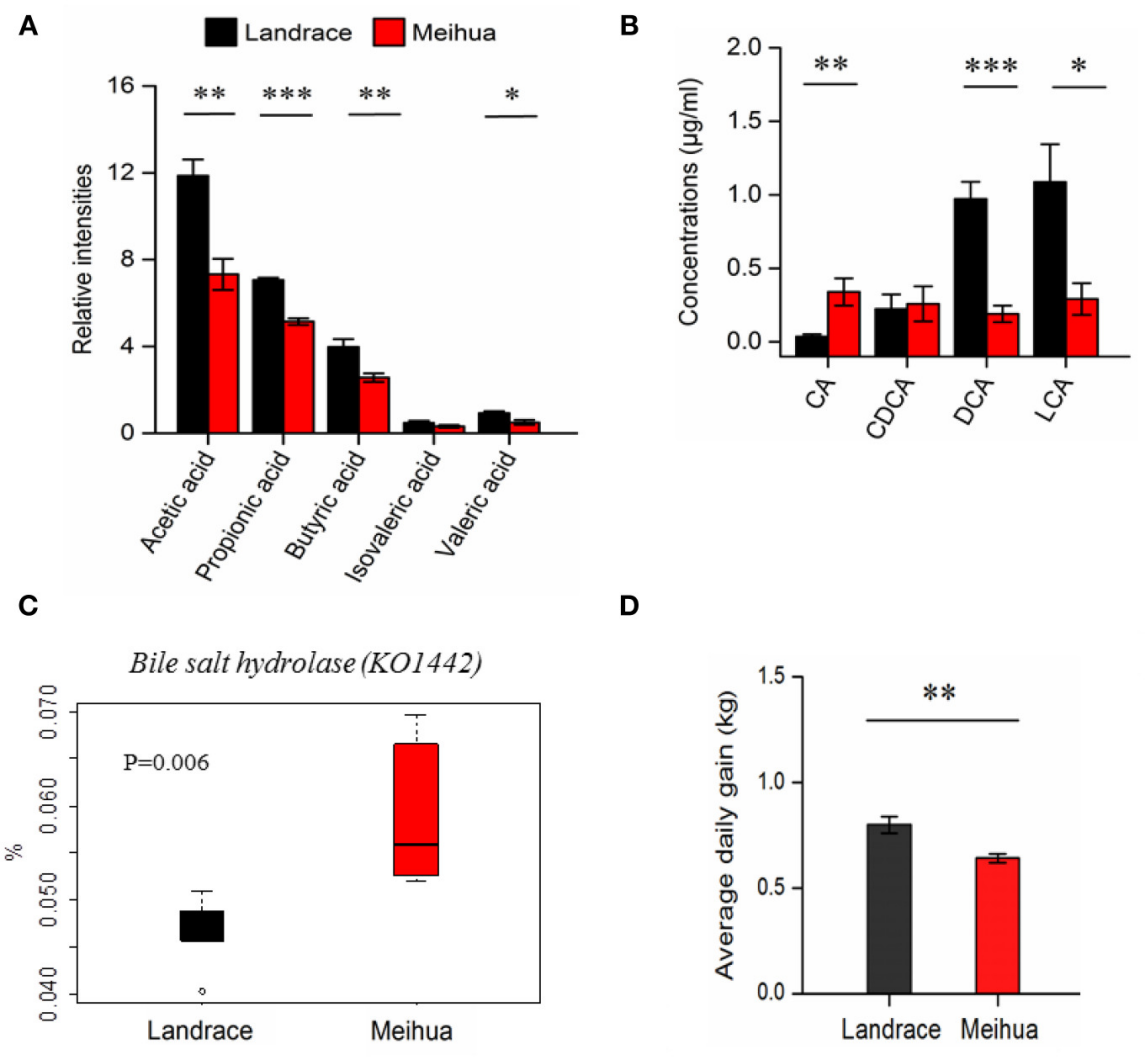
Quantification of short chain fatty acids (SCFAs) and bile acids in the colon lumen using the targeted metabolomics approach and average daily weight gain of piglets. **(A)** The relative levels of SCFAs in the colonic contents samples from Landrace and Meihua piglets. **(B)** The absolute levels of bile acids in the colonic contents samples from Landrace and Meihua piglets. CA, cholic acid; DCA, deoxycholic acid; CDCA, chenodexycholic acid; LCA, lithocholic acid. Error bar indicated the mean value ± SEM of each experimental group. **(C)** The relative abundance of bile salt hydrolase (K01442) from the colonic microbiota in Landrace and Meihua piglets. Based on the PICRUSt prediction and KEGG analysis, the KO term named K01224 was enriched in the pathways of primary bile acid biosynthesis and secondary bile acid biosynthesis. **(D)** Average daily weight gain of Landrace and Meihua piglets between days 30 and 65 of age. Results are shown as mean ± SE. ^*^P < 0.05, ^**^P < 0.01, and ^***^P < 0.001, respectively.

In order to validate differences in the gut metabolome between the two pig breeds, we used a targeted metabolomics approach to quantify the concentrations of some microbiota-derived metabolites (bile acids and SCFAs) in the colon lumen. Based on the GC-MS results, we found that levels of acetic acid, propionic acid, butyric acid, and valeric acid were much higher in the colon lumen of Landrace compared with Meihua (*P* < 0.05), but their levels of isovaleric acid did not differ (Figure 2A). The ratio of each SCFAs component was recorded as followed: 48.8 and 46.2% for acetic acid, 29.0 and 32.5% for propionic acid, 16.4 and 16.2% for butyric acid, 2.0 and 2.0% for isovaleric acid and, 3.8 and 3.1% for valeric acid in Landrace and Meihua, respectively (Figure S2B).

Based on the untargeted metabolomics approach, metabolites from bile acid metabolism were different between these two pig breeds (Table 1). Due to these differences, we further quantified the primary and secondary bile acids by LC-MS/MS. The primary bile acid (CA), which was derived from the conjugated endogenous bile acids by specific gut bacteria, was much higher in Meihua than Landrace (*P* < 0.05), while the secondary bile acids (DCA and LCA) derived from the primary bile acids were significantly lower in Meihua compared with Landrace, particularly for DCA (*P* < 0.05) (Figure 2B). As expected, the average daily weight gain of Landrace piglets during days 30–65 was higher than that of Meihua piglets (*P* < 0.05) (Figure 2D).

### Higher expression of SCFA transporters and receptor genes but lower expression of bile acid receptor genes found in the colon mucosa of Landrace piglets

**Figure 3:**
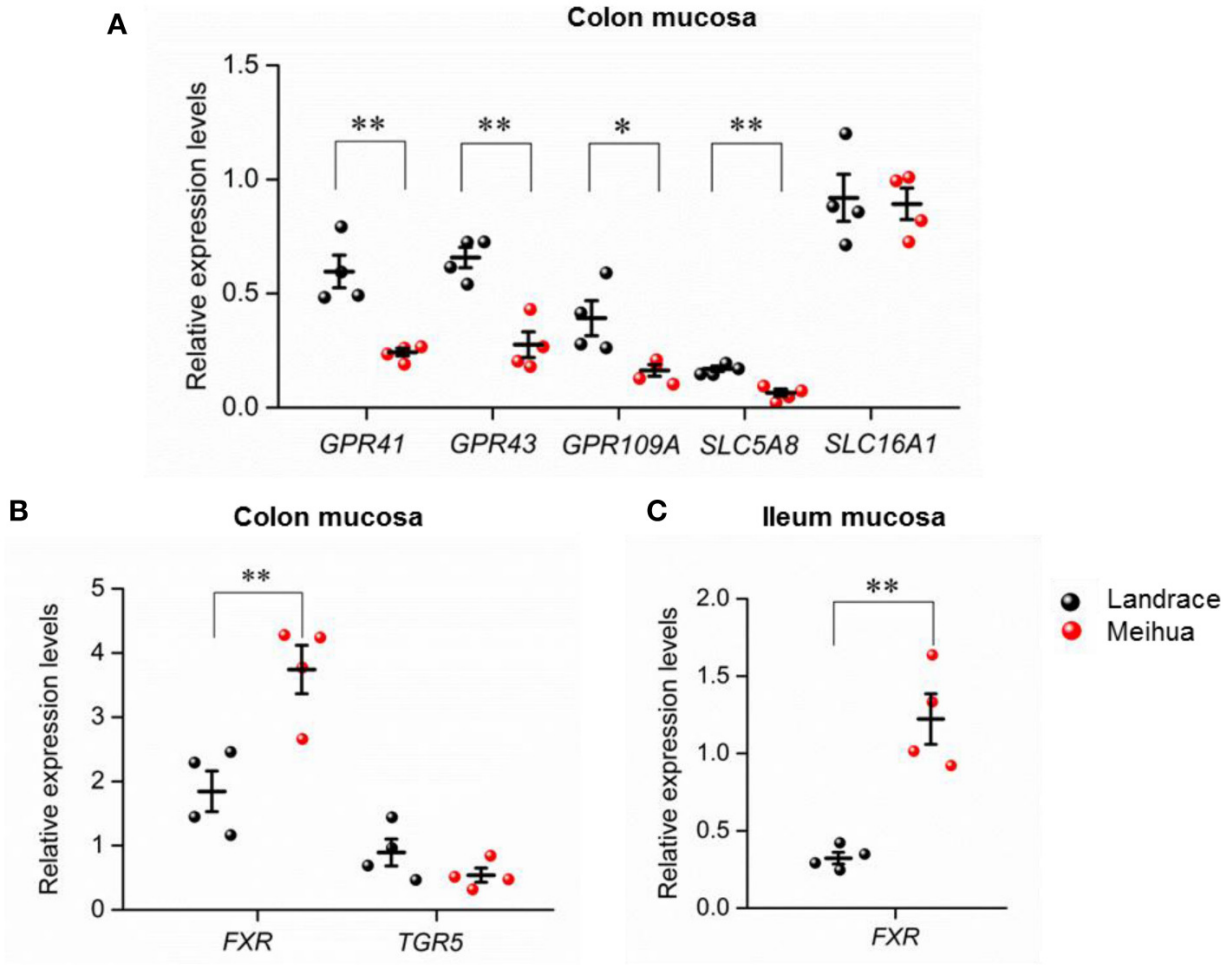
Expression levels of genes related to SCFAs and bile acids in different tissues of Landrace and Meihua piglets. **(A)** Comparison of expression levels of SCFA-receptor genes in colon mucosa between Landrace and Meihua piglets. GPR41, GPR43, and GPR109A are receptors of SCFAs, while SLC5A8 and SLC16A1 are transporters of SCFAs. Comparison of expression levels of bile acid-related genes in colon mucosa **(B)** and ileum mucosa tissues **(C)** between Landrace and Meihua piglets. FXR and TGR5 are both receptors of bile acids. ^*^P < 0.05, ^**^P < 0.01, and ^***^P < 0.001, respectively. Results are shown as mean ± SE.

To further explore whether SCFA levels in the colon lumen were associated with the expression of transporter and receptor genes of SCFAs in piglets, qRT-PCR was performed on the colon mucosa tissues of the piglets. As expected, relative mRNA expression of three receptor genes activated by SCFAs (*GPR41, GPR43*, and *GPR109A*) (Kasubuchi et al., 2015), were higher in colon tissues of Landrace (Figure 3A). In addition, one of the transporters of SCFAs, *SLC5A8*, was also highly expressed in Landrace when compared to Meihua piglets (Figure 3A). The result indicated that the high levels of SCFAs in the colon lumen of Landrace might be positively correlated with the expression of *GPR41* and *SLC5A8* in colon mucosa.

For bile acids in the colon lumen, Landrace piglets had higher levels of the secondary bile acids, LCA, and DCA, when compared to Meihua piglets. Accordingly, we found that the expression levels of *FXR*, but not *TGR5*, were up-regulated in colon and ileum mucosa of Meihua, the opposite pattern of the levels of secondary bile acids in colon content (Figures 3B,C).

### Differences in colonic luminal microbiome between Landrace and Meihua piglets

To further assess whether differences in gut microbiota are the causal factor for the differences in colonic luminal metabolomes between Landrace and Meihua piglets, high-throughput sequencing was performed to analyze 16S rRNA of bacteria from both pig breeds. A total of 792,455 quality-filtered sequences were obtained with an average of 56,604 sequences per sample in colonic microbiota. Then, four alpha diversity indices including observed species, Chao1, PD whole tree, and Shannon, were estimated. Community diversity index (PD whole tree) and community richness index (Chao1) of colonic microbes in Landrace piglets were significantly higher than those in Meihua piglets (*P* < 0.05, Table S4). The PCoA indicated that groups were not distinctly clustered separately in distribution of microbiota at the colonic contents (Figure S3). The relationships of gut microbiota between groups were calculated using permutational multivariate analysis of variance (PERMANOVA) based on Bray–Curtis distance. Results showed that community structures in the colon were not significantly different between Landrace and Meihua piglets at the genus level (*P* > 0.05, Table S4).

**Figure 4:**
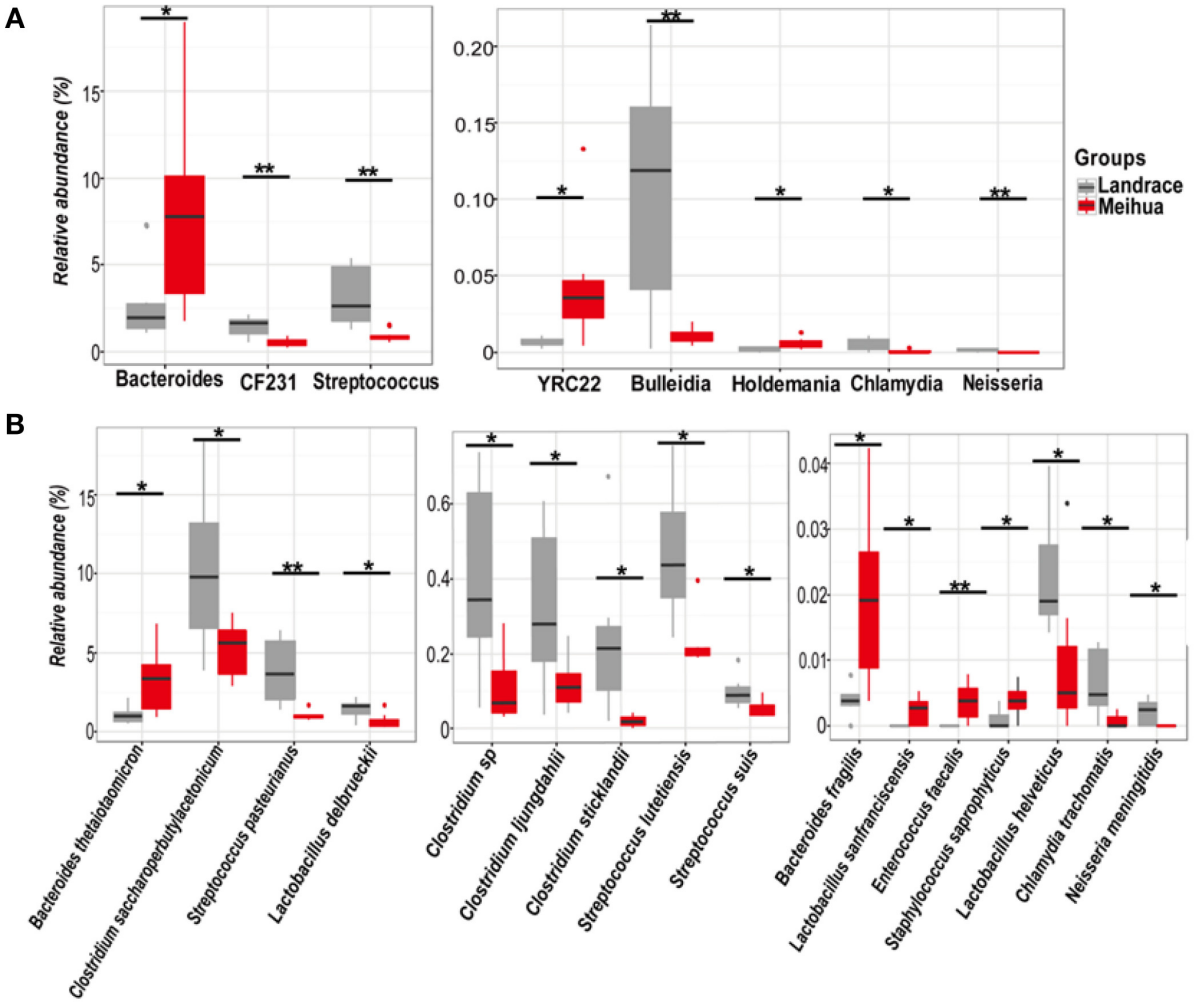
Relative abundances of bacterial genera **(A)** and species **(B)** in the colon differed significantly between Landrace (gray) and Meihua (red) piglets. ^*^P < 0.05, and ^**^P < 0.01.

Firmicutes and Bacteroidetes were the most predominant phyla in the colons of both Landrace and Meihua piglets, comprising more than 88% of the total sequences. There was no significant difference in bacterial phyla in the colonic contents between two breeds. At the genus level, the most abundant genus in both pig breeds was *Prevotella*, followed by the genera *Lactobacillus, Bacteroides*, and *Streptococcus*. The proportion of *Streptococcus, CF231, Bulleidia*, and *Chlamydia* in Landrace were 2.6, 1.8, 9.2, and 6.6-fold higher than those in Meihua, respectively (*P* < 0.05) (Figure 4A). The proportion of *Bacteroides, YRC22*, and *Holdemania* in Landrace were 0.8, 0.9, and 0.7-fold lower than those in Meihua, respectively (*P* < 0.05) (Figure 4A). The genus *Neisseria* was found in Landrace piglets, but was absent from Meihua piglets.

We further assessed differences in the bacterial community at the species level using the BLAST algorithm to align the assembled 16s rRNA sequences to the bacterial genome and parsed the alignment results using the filtered setting (see section Materials and Methods). A combined total of 217 bacterial species were identified in the colons of two pig breeds. *Prevotella dentalis* and *Prevotella melaninogenica* were the most common species and both accounted for more than 10% of all colonic microbes in Landrace and Meihua piglets. Significant differences in four *Clostridium* species were observed between the two breeds. The relative abundances of *Clostridium saccharoperbutylacetonicum, Clostridium* sp., *Clostridium ljungdahlii*, and *Clostridium sticklandii* were higher in Landrace piglets than Meihua piglets (*P* < 0.05) (Figure 4B). The relative proportions of *Streptococcus pasteurianus, Streptococcus lutetiensis, Streptococcus suis, Lactobacillus helveticus*, and *Chlamydia trachomatis* were also higher in Landrace piglets than Meihua piglets (*P* < 0.05) (Figure 4B). However, *Bacteroides thetaiotaomicron, Bacteroides fragilis, Lactobacillus sanfranciscensis, Enterococcus faecalis*, and *Staphylococcus saprophyticus* were lower in Landrace piglets than Meihua piglets (*P* < 0.05) (Figure 4B). The species *Neisseria meningitides* exists in Landrace but was not present in Meihua piglets.

The PICRUSt analysis was used for predicting the potential functions of the intestinal microbiota. Based on the level 2 of KEGG Pathway analysis, it showed that the colon luminal microbial carbohydrate metabolism pathway in Meihua piglets was more abundant than in Landrace piglets (Figure S4A). Furthermore, pathway enrichments at KEGG level 3 also showed that Meihua piglets had higher enrichment of the pathways involved in carbohydrate metabolism, including (1) galactose, fructose, and mannose metabolism; (2) other glycan degradation; and (3) starch and sucrose metabolism (Figure S4B). Less obviously but significantly, the abundance of pathways for biosynthesis of primary and secondary bile acids in Meihua piglets were higher than in Landrace piglets (Figure S4B). In addition, more enrichment was detected for sphingolipid metabolism and the insulin signaling pathway in Meihua piglets (Figure S4B). However, colonic microbiota in Landrace had higher enrichment of pathways involved in branched chain amino acid degradation, butanoate metabolism, as well as in flagellar assembly, secretion system, bacterial motility proteins, and bacterial chemotaxis processes (Figure S4B).

Finally, we compared the abundance of bile salt hydrolase (KO1442) in the colonic microbiota between the two pig breeds. Bile salt hydrolase is the key enzyme involved in metabolism of primary bile acids. We observed that the abundance of bile salt hydrolase (KO1442) from colonic microbiota in Landrace piglets was significantly lower compared to Meihua piglets (*P* < 0.05) (Figure 2C). This was consistent with the higher level of the unconjugated primary bile acid CA in Meihua piglets.

### Correlation between microbial communities and their metabolites

**Figure 5:**
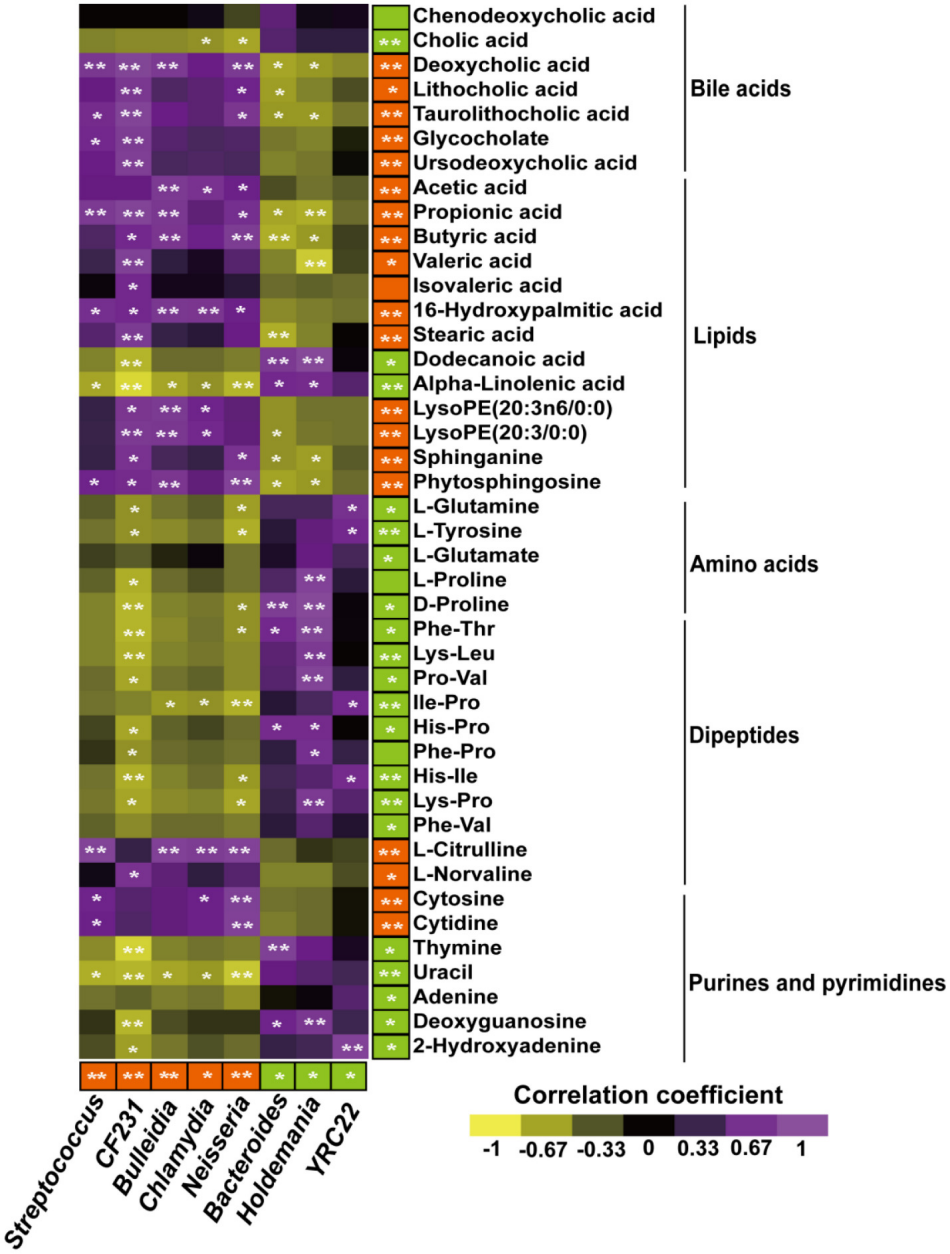
Pearson's correlation analysis of colonic metabolites and colonic bacterial genus-level taxa in Landrace and Meihua samples. Bacterial genera and colonic metabolites enriched in Landrace or Meihua samples are colored red and green, respectively. An asterisk in the colored box indicates that a genera or metabolite is significantly enriched in Landrace (red) or Meihua (green) samples. Correlations with *P* ≤ 0.05 are shown. Yellow represents a significant negative correlation (*P* < 0.05), purple represents a significant positive correlation (*P* < 0.05), and black represents no significant correlation (*P* > 0.05). Orange color with ^*^represents a higher value in Landrace (*P* < 0.05), and ^**^P < 0.01.

Correlations between metabolites and 8 genera (Figure 5) or 16 species (Figure S5) of bacteria with significant differences between Landrace and Meihua piglets were obtained via Pearson's correlation analysis. As shown in Figure 5, the relative higher abundances of *Streptococcus, CF231, Bulleidia, Chlamydia*, and *Neisseria* were positively associated with higher concentrations of microbial metabolites in Landrace, including bile acids, SCFAs, and lipids (*P* < 0.05). The relative lower abundances of *Bacterioides, Holdemania*, and *YRC22* were negatively associated with the higher concentrations of bile acids, SCFAs, and lipids in Landrace, when compared to Meihua piglets (*P* < 0.05).

Furthermore, the relative higher abundances of 16 bacterial species in Landrace (Figure S5), including *C. saccharoperbutylacetonicum, Clostridium* sp., *C. ljungdahlii, C. sticklandii, S. pasteurianus, S. lutetiensis, S. suis, Lactobacillus delbrueckii, L. helveticus*, and *C. trachomatis* were positively correlated with the higher concentrations of bile acids, SCFAs, and lipids (*P* < 0.05). The relative lower abundances of *N. meningitides, B. thetaiotaomicron, B. fragilis, S. saprophyticus, E. faecalis*, and *L. sanfranciscensis* were negatively correlated with lower concentrations of metabolites such as amino acids, dipeptides, purines, and pyrimidines, when compared to Meihua piglets (*P* < 0.05) (Figure S5).

## Discussion and conclusion

The host gut–microbial relationship is of great importance to host phenotype, physiology, and health status (Lalles, 2016). However, whether or not the development of piglets with different genetic backgrounds will be influenced by differences in the intestinal microbiome and its metabolites requires further investigations. In-depth analyses of gut microbiome and metabolic activities using “omics” approaches will help identify microbial biomarkers that facilitate the animal production and phenotype identification. Here, we investigated the composition of the microbiome and its metabolites in the colon contents of two pig breeds, using a combination of 16S rRNA gene high-throughput sequencing and MS-based metabolomics techniques. We found that the microbiome and metabolome in the colon lumen were significantly different between Meihua and Landrace piglets, breeds with varied growth rates (Figure 2D). The relative abundances of 8 genera and 16 species of bacteria differed significantly between Landrace and Meihua piglets. A total of 401 metabolites in the colon lumen differed significantly in relative levels between the two pig breeds. Seventy of these metabolites were definitively identified, including: bile acids, dipeptides, sphingolipids, amino acids, nucleotides, and many hydrophilic molecules. Differences between concentrations of bile acids and SCFAs in the colon lumen were further validated through targeted metabolomics approaches as well as gene expression of their transporters and receptors determined by qPCR.

The SCFAs, one of the most abundant microbial metabolites, are mainly produced by colonic bacteria through fermentation of carbohydrates, but a small portion (~5%) are produced from protein or amino acids that are unabsorbed or undigested in the small intestine. Evidence has shown that the production of intestinal SCFAs can be influenced by dietary factors and food intake patterns (Rios-Covian et al., 2016). Whether the genetic variance of pigs or exposure to the pre-weaning diet led to differences in colonic SCFAs levels needs to be investigated. Our results have shown that the concentrations of most SCFAs, including acetic acid, propionic acid, butyric acid, and valeric acid, were higher in Landrace than in Meihua piglets. Our results were similar to previous reports that Bama mini-pig (a fatty-type Chinese local pig strain) had lower concentrations of total SCFAs in colon content than Landrace (Jiang et al., 2016). Previous study has also demonstrated that Chinese native Lantang pigs produced more SCFAs in the large intestine than Duroc pigs (Cheng et al., 2017). It is well known that acetate, propionate, and butyrate account for more than 90% of the total SCFAs in the colon (Rios-Covian et al., 2016). Similarly, our results indicate that the proportions of these three SCFAs in the colon of Landrace and Meihua piglets reached 94.2 and 94.9%, respectively (Figure S2B).

The higher levels of SCFAs in Landrace were also supported by an increased abundance of SCFAs-producing bacteria. Even though we did not observe differences between bacterial phyla from the colonic lumen of Landrace and Meihua piglets, we found that *Clostridium* species like *C. saccharoperbutylacetonicum, Clostridium* sp., *C. ljungdahlii*, and *C. sticklandii* were all more abundant in colonic microbiota of Landrace piglets. Notably, differences in genera and species of bacteria are believed to contribute to specific metabolic functions (Bauer et al., 2016). Bacterial genera such as the *Clostridium* clusters IV and XIVa of Firmicutes, including species of *Eubacterium, Roseburia, Faecalibacterium*, and *Coprococcus*, are involved in SCFAs production (Nicholson et al., 2012; Van den Abbeele et al., 2013), and might thus impact swine health and development (Park et al., 2014).

Using KEGG pathway analysis, we found that carbohydrate metabolism by microbes was enriched in the colon tract of Meihua piglets, and intermediate metabolites involved in carbohydrate metabolism were identified by metabolomic analysis. We also found that ATP levels in the colon content of Meihua piglets were significantly higher than in Landrace piglets (Figure S2A), which indicates that the ability of gut microbiota to metabolize carbohydrates may not indicate high efficiency in production of SCFAs.

Microbial-derived SCFAs are almost totally absorbed by colonocytes, either through diffusion or through transport by monocarboxylate transporters like SLC16A1 and SLC5A8 (Ganapathy et al., 2013), with a small portion (<10%) excreted in the feces (Boets et al., 2015). The transporter SLC5A8, which can transport a variety of SCFAs, mediates beneficial effects of SCFAs especially when the concentration of SCFAs is low in the colon lumen (Ganapathy et al., 2013). In our study, the expression of *SLC5A8*, but not *SLC16A1*, in the colon mucosa was found to be higher in Landrace than Meihua piglets, which is consistent with the higher concentration of SCFAs mentioned above. Our results were also in accordance with a previous study demonstrating lower levels of mRNA and proteins of SLC5A8 in germ-free mice in contrast to wild-type mice (Cresci et al., 2010). Importantly, the result of *SLC5A8* expression was in agreement with the higher richness and diversity of colon luminal microbiota in Landrace compared to Meihua piglets, as shown by 16S sequencing.

SCFAs are not only the critical energy sources for colonocytes (Xiong et al., 2004), but also serve as key regulators of the intestinal epithelial barrier and gut immunity (Rios-Covian et al., 2016). These functions of SCFAs may facilitate better growth performance in Landrace compared to Meihua piglets. In addition, the biological functions of SCFAs depend on their specific receptors (GPR41, GPR43, and GPR109A) to affect host physiological processes, including regulation of energy metabolism in mammals (Kasubuchi et al., 2015; Hu et al., 2016; Rios-Covian et al., 2016). In our study, Landrace had higher expression of *GPR41* in the colon mucosa compared to Meihua piglets. This result was in accordance with the higher levels of lipid and lipid-like molecules, and lower concentrations of amino acids and dipeptides detected in the colon contents of Landrace by untargeted metabolomics analyses. However, another study has shown that the fatty-type Bama mini-pig expressed higher mRNA levels of *GPR41* and *GPR43* in colonic tissue compared to lean-type Landrace (Jiang et al., 2016). The discrepancy between the previous results and ours may involve differences in fatty-type pig breed used, experimental design, dietary composition, and sampling sites.

Apart from the differences in SCFAs production and expression of *SLC5A8* and *GPR41* between Landrace and Meihua piglets, another important finding of our study was the significant difference of secondary bile acids between the two pig breeds. During metabolism of bile acids, taurine- or glycine-conjugated bile acids were found to escape from the distal ileum when reabsorbed to enterohepatic circulation. However, before these bile acids escape, they are deconjugated by bile salt hydrolase secreted by gut microbiota including *Lactobacillus, Bifidobacteria, Enterobacter, Bacteroides*, and *Clostridium* (Wahlstrom et al., 2016). Primary bile acids are then oxidized or dehydroxylated by other microbiota-produced enzymes like hydroxy-steroid dehydrogenases (Ridlon et al., 2006) from bacteria involved in secondary bile acid fermentation including *Clostridium* (clusters XIVa and XI) and *Eubacterium* (Wahlstrom et al., 2016). Here, we showed that the abundance of bacterial genes (bile salt hydrolase, KO1442) related to secondary bile acid biosynthesis was higher in the colonic lumen of Meihua piglets, while the absolute quantification of secondary bile acids (DCA and LCA) in the colon contents was lower. Our results were in accordance with the previous results that a higher amount of bile acids was associated with lower butyrate concentrations in the rat cecum (Islam et al., 2011), probably due to inhibition of the proliferation of butyrate-producing bacteria or of the butyrate synthesis metabolic pathways (Ha et al., 2014).

The mRNA expression of bile acid receptor *FXR*, which is activated by unconjugated primary and secondary bile acids and inhibited by conjugated bile acids (Wahlstrom et al., 2016), was significantly higher in the colon and ileum of Meihua piglets than those of Landrace. Previous studies also demonstrated that the inhibition of FXR by gut microbiota was tightly linked to decreased hepatic lipid synthesis (Zhang et al., 2016) and alleviated obesity phenotypes (Li F. et al., 2013). Compared to wild type mice, FXR-deficient mice have a higher relative ratio of *Firmicutes* to *Bacteroidetes* in the intestine, as well as reduced obesity (Parseus et al., 2017). In addition, bile acids in the gut lumen can also directly regulate microbiotic functioning by serving as an antibiotic for bile sensitive bacteria or as a promoter for bile acid-metabolizing bacterial communities (Hofmann and Eckmann, 2006; Wahlstrom et al., 2016). In the present study, a higher abundance of *Bacteroides* spp., which are advantageous to bile acid biosynthesis (Wahlstrom et al., 2016), were found in the colon lumen of Meihua compared to Landrace piglets.

By performing Pearson's correlation analyses, the relative abundances of bacteria at either the genus or species levels were found to be closely associated with the concentrations of specific microbial metabolites in the colonic lumen. For example, Firmicutes species like *Clostridium* sp., *L. delbrueckii, L. helveticcus*, and *Streptococcus lutetiensis*, were positively correlated with secondary bile acids as well as SCFAs. The diversity of gut microbiota species and the abundance of SCFA-producing bacteria are believed to be associated with energy harvesting and body weight. Recent study has also shown that there is a possible link between the intestinal microbiota and feed efficiency in pigs (McCormack et al., 2017). Landrace pigs are known for good growth performance and a high ratio of lean meat (Li Z. et al., 2013). In accordance with this characteristic, we found that the average daily weight gain of Landrace piglets was higher than that of Meihua piglets. Collectively, the higher production of SCFAs and lower amount of secondary bile acids in response to differences in the colonic microbiome may be positively correlated with a faster growth rate in Landrace pigs. Further mechanisms underlying inheritance, diets, and environment in regulating host phenotypes should be explored, focusing on SCFAs and bile acids as well as their receptors.

In conclusion, the microbial communities and metabolome profiles in the colon lumen were influenced by host genetics, and displayed significant differences between the fatty-type Meihua and the lean-type Landrace piglets. In the present study, significant differences in the production of SCFAs and secondary bile acids, as well as expression of their receptors, in the colon between Landrace and Meihua piglets were clearly demonstrated for the first time. The integration of results from the gut luminal metabolome and microbiome not only provide an understanding of the metabolic differences of gut microbiota between these two pig breeds, but also may have great potential as biomarkers for some metabolic diseases in human.

## Author contributions

ZJ and ZhuC conceived and designed the experiments, SY, CZ, and TY analyzed the data and drafted the manuscript, WH, JH, QK, JS, ZhoC, QL, and SW performed the experiments and analyzed the data.

### Conflict of interest statement

The authors declare that the research was conducted in the absence of any commercial or financial relationships that could be construed as a potential conflict of interest.
