# Supplementary material for: Studying the Differences of Bacterial Metabolome and Microbiome in the Colon between Landrace and Meihua Piglets
Source: Front Microbiol. 2017 Sep 21;8:1812. doi: 10.3389/fmicb.2017.01812 (PMC5613163; doi:10.3389/fmicb.2017.01812)
Supplement: Supplementary file 1 [file Presentation1.PDF]

Supplementary Information for:

Distinct differences of bacterial metabolome and microbiome in the colon between Landrace and Meihua piglets

Shijuan Yan[1,#], Cui Zhu[1,#], Ting Yu[1,#], Wenjie Huang[1], Jianfeng Huang[1,3], Qian Kong[1], Jingfang Shi[1], Zhongjian Chen[1], Qinjian Liu[1], Shaolei Wang[1], Zongyong Jiang[1,2,*], and Zhuang Chen[1,*]

[1] Agro-biological Gene Research Center, Guangdong Academy of Agricultural Sciences, Guangzhou, China

[2] Ministry of Agriculture Key Laboratory of Animal Nutrition and Feed Science (South China), Institute of Animal Science, Guangdong Academy of Agricultural Sciences, Guangzhou, China

[3] Brain Science Institute, South China Normal University, Guangzhou, China

[*]Address correspondence to Dr. Zhuang Chen (chenzhuang@agrogene.ac.cn) or Dr. Zongyong Jiang (jiangzy@gdaas.cn)

[#]These authors contributed equally to this work.

This file include:

Supplementary Fig. 1—5

Supplementary Table 1—4

Supplementary Data 1

Supplementary Figures

Fig. S1. Metabolic pathway enrichment analysis of the identified metabolites showing significant difference between Landrace and Meihua piglets. Overview of metabolites in the colon lumen that were enriched in Landrace piglets compared to Meihua piglets. The x-axis represents the pathway impact, and y-axis represents the pathway enrichment. Larger sizes and darker colors represent higher pathway enrichment and higher pathway impact values, respectively.

Fig. S2. Energy metabolism of colonic microbiota. (a) Concentration of adenosine phosphate in colon content. ** represented $P< 0.01$. (b) The ratio of each SCFA component in the colonic contents samples from Landrace and Meihua piglets.

Fig. S3. Principal Coordinate Analysis (PCoA) of the bacterial composition in the colon lumen of piglets. The red dots represented 7 Meihua piglets, and the black dots represented 7 Landrace piglets.

Fig. S4. Extended error bar plots identifying significant differences in pathway enrichment from bacterial sequences at KEGG level 2 (a) and KEGG level 3 (b). Note: bacterial sequences from Landrace (black) and Meihua (red) piglets were compared. Corrected *P*-values are shown at right.

Fig. S5. Pearson's correlation analysis of colonic metabolites and colonic bacterial species-level taxa. Bacterial species and colonic metabolites enriched in Landrace or Meihua samples are colored red and green, respectively. An asterisk in the colored box indicates that a taxon or metabolite is significantly enriched in Landrace (red) or Meihua (green) samples. Note: Correlations with $P \leq 0.05$ are shown. Yellow represents a significant negative correlation ($P < 0.05$), purple represents a significant positive correlation ($P < 0.05$), and black represents no significant correlation ($P > 0.05$). Orange color with * represents a higher value in Landrace ($P < 0.05$), and ** represents $P < 0.01$. Green color with * represents a higher value in Landrace ($P < 0.05$), and ** represents $P < 0.01$.

Fig. S1

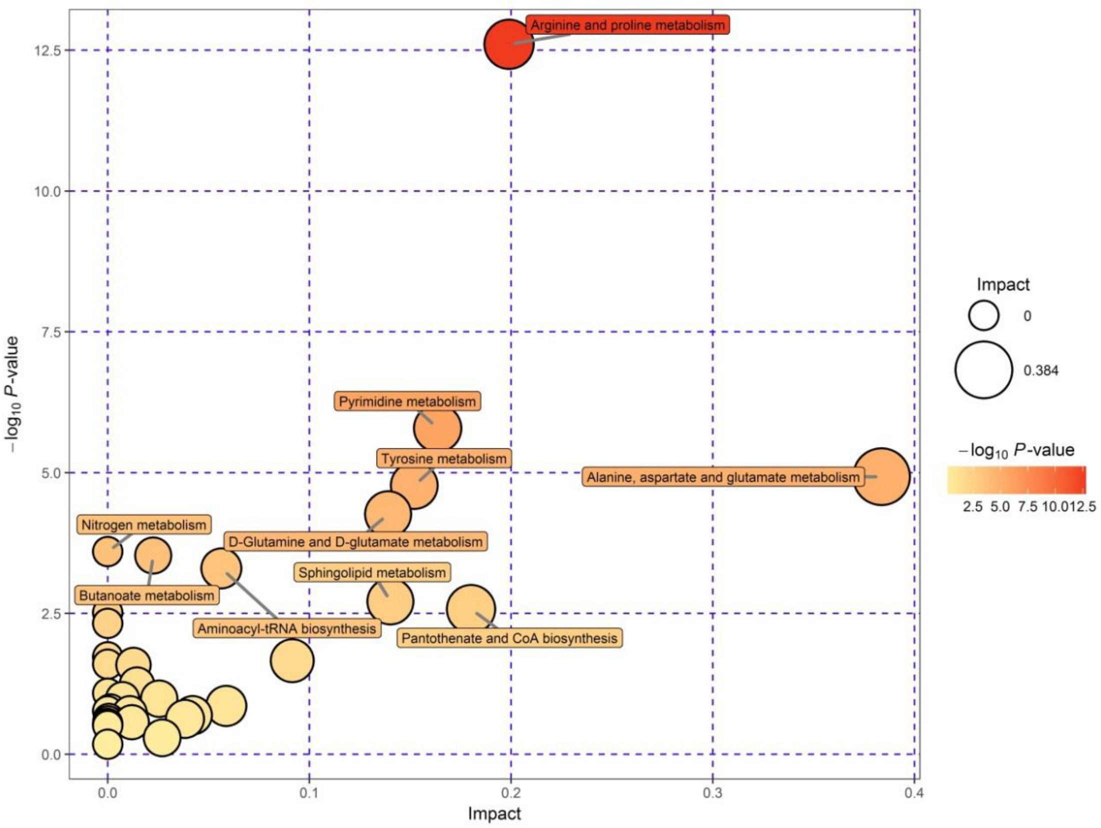

Arginine and proline metabolism
Pyrimidine metabolism
Tyrosine metabolism
Alanine, aspartate and glutamate metabolism
Nitrogen metabolism
D-Glutamine and D-glutamate metabolism
Sphingolipid metabolism
Butanoate metabolism
Aminoacyl-tRNA biosynthesis
Pantothenate and CoA biosynthesis
12.5
10.0
7.5
5.0
2.5
0.0
0.0
0.1
0.2
0.3
0.4
Impact
$-\log_{10}$ P-value
Impact
0
0.384
$-\log_{10}$ P-value
2.5 5.0 7.5 10.0 12.5

Fig. S2

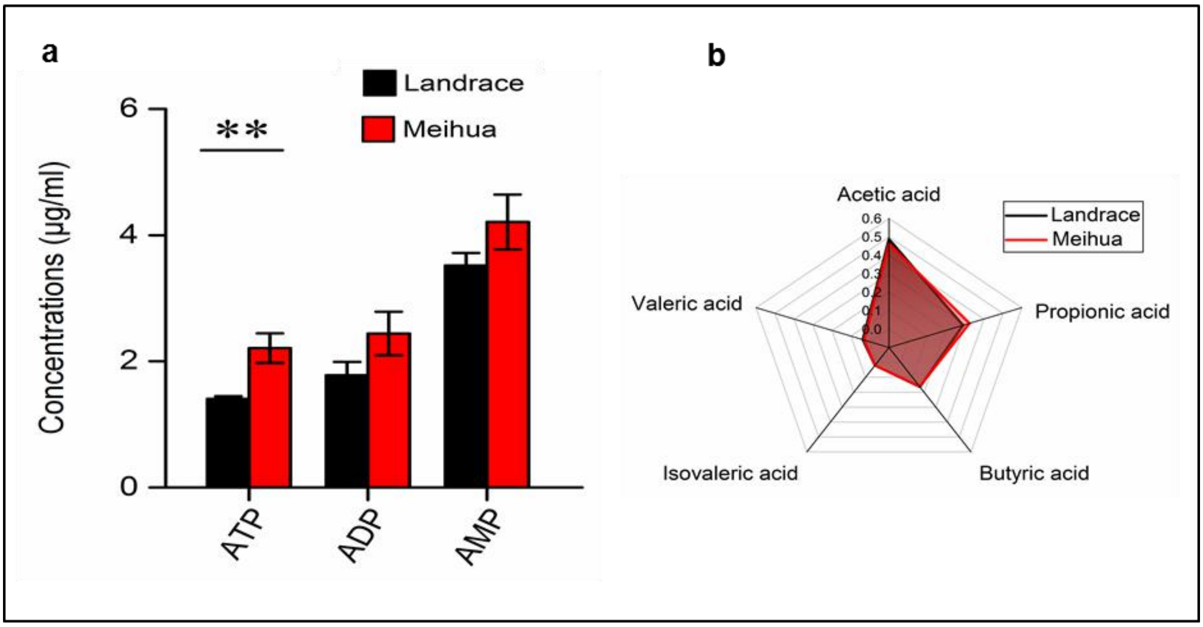
a
Landrace
Meihua
Concentrations (μg/ml)
6
4
2
0
**
ATP
ADP
AMP
b
Acetic acid
Propionic acid
Butyric acid
Isovaleric acid
Valeric acid
Landrace
Meihua
0.6
0.5
0.4
0.3
0.2
0.1
0.0

Fig. S3

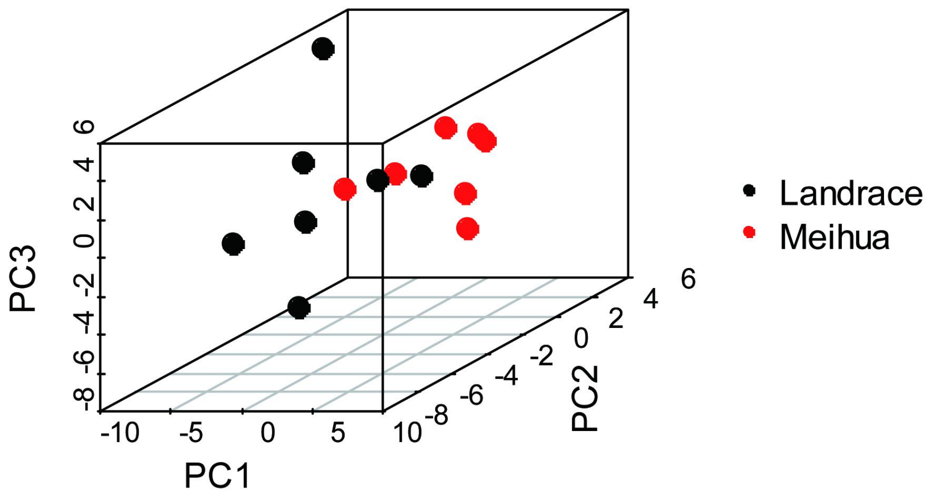

Fig. S4

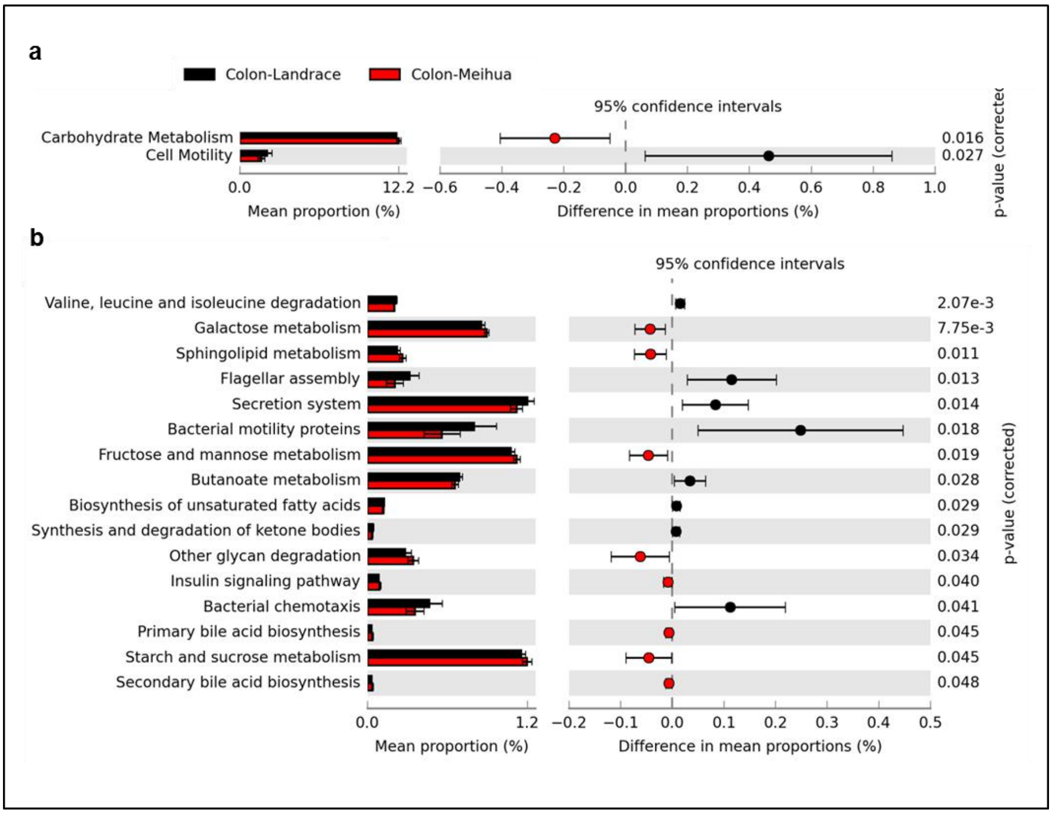

a
Colon-Landrace
Colon-Meihua
95% confidence intervals
Carbohydrate Metabolism
Cell Motility
0.016
0.027
0.0
12.2
−0.6
−0.4
−0.2
0.0
0.2
0.4
0.6
0.8
1.0
Mean proportion (%)
Difference in mean proportions (%)
p-value (correctec
b
95% confidence intervals
Valine, leucine and isoleucine degradation
Galactose metabolism
Sphingolipid metabolism
Flagellar assembly
Secretion system
Bacterial motility proteins
Fructose and mannose metabolism
Butanoate metabolism
Biosynthesis of unsaturated fatty acids
Synthesis and degradation of ketone bodies
Other glycan degradation
Insulin signaling pathway
Bacterial chemotaxis
Primary bile acid biosynthesis
Starch and sucrose metabolism
Secondary bile acid biosynthesis
2.07e-3
7.75e-3
0.011
0.013
0.014
0.018
0.019
0.028
0.029
0.029
0.034
0.040
0.041
0.045
0.045
0.048
0.0
1.2
−0.2
−0.1
0.0
0.1
0.2
0.3
0.4
0.5
Mean proportion (%)
Difference in mean proportions (%)
p-value (corrected)

Fig. S5

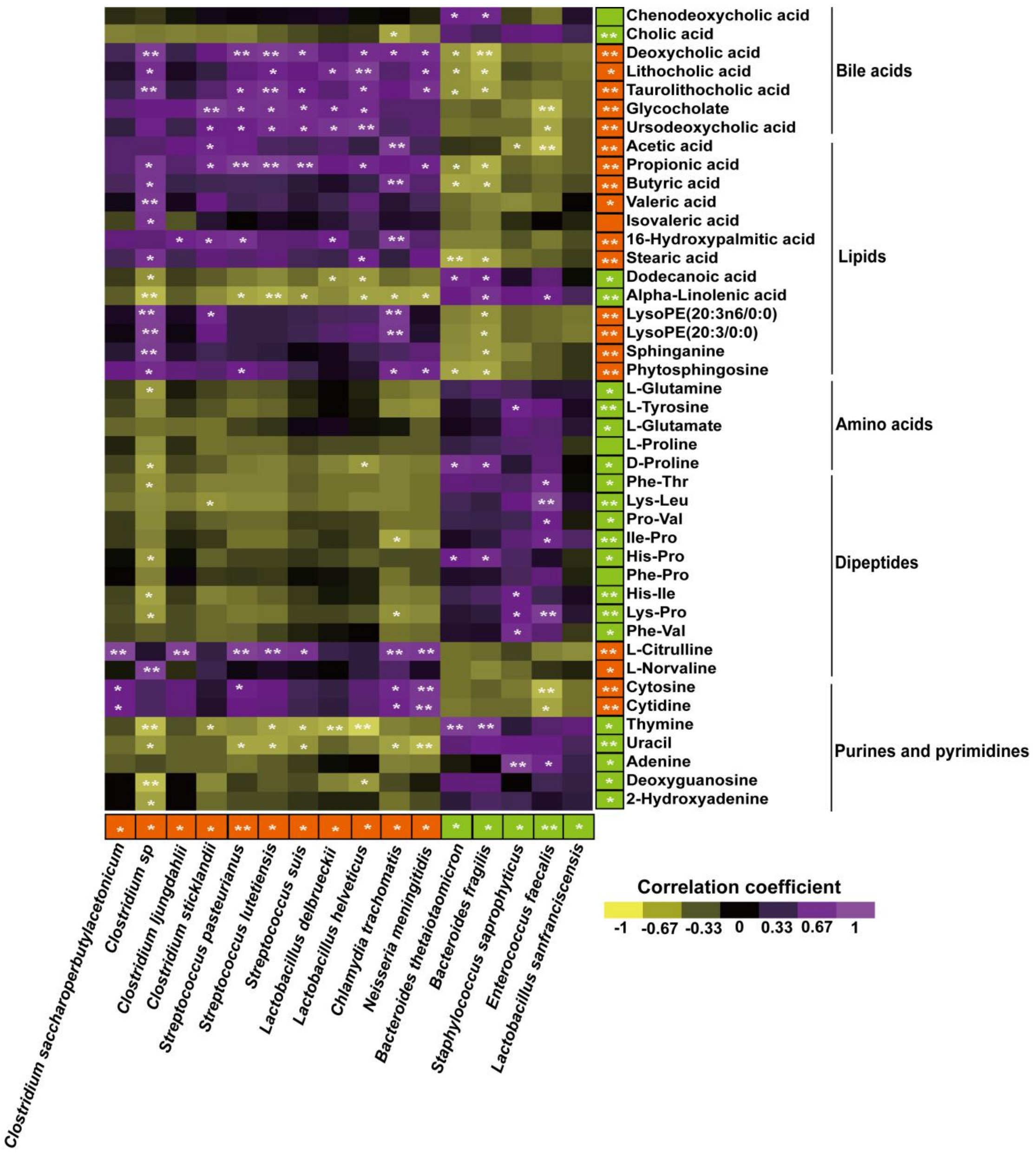
Chenodeoxycholic acid
Cholic acid
Deoxycholic acid
Lithocholic acid
Taurolithocholic acid
Glycocholate
Ursodeoxycholic acid
Acetic acid
Propionic acid
Butyric acid
Valeric acid
Isovaleric acid
16-Hydroxypalmitic acid
Stearic acid
Dodecanoic acid
Alpha-Linolenic acid
LysoPE(20:3n6/0:0)
LysoPE(20:3/0:0)
Sphinganine
Phytosphingosine
L-Glutamine
L-Tyrosine
L-Glutamate
L-Proline
D-Proline
Phe-Thr
Lys-Leu
Pro-Val
Ile-Pro
His-Pro
Phe-Pro
His-Ile
Lys-Pro
Phe-Val
L-Citrulline
L-Norvaline
Cytosine
Cytidine
Thymine
Uracil
Adenine
Deoxyguanosine
2-Hydroxyadenine
Bile acids
Lipids
Amino acids
Dipeptides
Purines and pyrimidines
Clostridium saccharoperbutylacetonicum
Clostridium sp
Clostridium ljungdahlii
Clostridium sticklandii
Streptococcus pasteurianus
Streptococcus lutetiensis
Streptococcus suis
Lactobacillus delbrueckii
Lactobacillus helveticus
Chlamydia trachomatis
Neisseria meningitidis
Bacteroides thetaiotaomicron
Bacteroides fragilis
Staphylococcus saprophyticus
Enterococcus faecalis
Lactobacillus sanfranciscensis
Correlation coefficient
-1
-0.67
-0.33
0
0.33
0.67
1

Supplementary Tables

Table S1. Characteristic fragment ions of CA, LCA, DCA, CDCA standards and their optimized MS/MS conditions.

Metabolites	Q1	Q3	Declustering potential (ev)	Collision Energy (ev)
CA	406.9	289.3	-130	-59
		195.0	-130	-52
LCA	375.2	375.2	-130	-7
DCA	391.2	343.4	-130	-53
		327.2	-130	-53
CDCA	391.2	391.2	-130	-34

Note: CA, cholic acid; DCA, deoxycholic acid, CDCA, chenodexycholic acid; LCA, lithocholic acid.

Table S2. Sequences, product sizes, and Tm values of primers for targeted genes.

Genes	Primer sequence (5’-3’)	Tm (℃)	Product size (bp)	Accession No.
FXR	F: TATGAACTCAGGCGAATGCCTGCT	60	154	NM_001287412.1
	R: ATCCAGATGCTCTGTCTCCGCAAA			
TGR5	F: CCATGCACCCCTGTTGCT	60	67	XM_013984487.1
	R: GGTGCTGTTGGGTGTCATCTT			
GPR41	F: GCCCTTGCCCTTCATCTTCT	60	136	JQ776642.1
	R: CCGGGTCTTGTACCAGAGTG			
GPR43	F: CAGAGGCAAAGAGACCGAGG	60	83	NM_001278758.1
	R: TGGTGAAGTCAGAACTCGGC			
GPR109A	F: CGCGATTTCCCAGAACTTCC	59	138	AK397813.1
	R: AGCTTCAGACGCCTAGGAAC			
SLC5A8	F: GGCACTCGTTTGTGAAGCTG	60	132	XM_013998027.1
	R: ATCCGCCCTCCCAAACATTC			
SLC16A1	F: CATCAACTACCGACTTCTG	59	80	NM_001128445.1
	R: TACTGGTCTCCTCCTCTT			
β-actin[a]	F: TCTGGCACCACACCTTCT	59-60	174	XM_003124280.4
	R: TGATCTGGGTCATCTTCTCAC			

FXR: farnesoid X receptor, *TGR5* : Takeda G protein-coupled receptor 5, *GPR41*: G protein-coupled receptors 41, *GPR43:* G protein-coupled receptors 43, *GPR109A:* G protein-coupled receptors 109A, *SLC5A8:* Solute Carrier Family 5 Member 8, *SLC16A1*: Solute Carrier Family 16 Member 1. R: reverse; F: forward. [a] The Tm value of *β-actin* varied from 59 to 60℃ in each target gene expression assay as those of target genes changed.

Table S3. Characterized parameters for the metabolic pathway enrichment analysis of the identified metabolites with significant difference between Landrace and Meihua piglets.

Pathway	Total	Hits	Raw p	-LOG(p)	Impact
Arginine and proline metabolism	77	9	3.35E-06	12.606	0.19899
Pyrimidine metabolism	60	5	0.00306	5.7895	0.16351
Alanine, aspartate and glutamate metabolism	24	3	0.007244	4.9275	0.38367
Tyrosine metabolism	76	5	0.00847	4.7712	0.152
D-Glutamine and D-glutamate metabolism	11	2	0.01413	4.2595	0.13904
Nitrogen metabolism	39	3	0.027423	3.5964	0
Butanoate metabolism	40	3	0.029302	3.5301	0.02254
Aminoacyl-tRNA biosynthesis	75	4	0.036851	3.3009	0.05634
Sphingolipid metabolism	25	2	0.066363	2.7126	0.1402
Pantothenate and CoA biosynthesis	27	2	0.076018	2.5768	0.18014

Table S4. Richness and diversity indices estimated for colonic microbiota in Landrace and Meihua piglets.

	Richness indices		Diversity indices	
Item	Observed_species	Chao1	PD_whole_tree	Shannon
Landrace	7536.77	18379.14	377.35	10.05
Meihua	7179.11	16687.36	352.03	9.96
P value	0.18	0.04	0.036	0.35

Supplementary Data

Data S1. Detailed information about the 401 biomarker metabolites detected by LC-MS (ESI+) and LC-MS (ESI+). (See separate Excel file)
